# The Mutation Analysis of the AMT Gene in a Chinese Family With Nonketotic Hyperglycinemia

**DOI:** 10.3389/fgene.2022.854712

**Published:** 2022-05-12

**Authors:** Bing-bo Zhou, Ling Hui, Qing-hua Zhang, Xue Chen, Chuan Zhang, Lei Zheng, Xuan Feng, Yu-pei Wang, Zhong-jun Ding, Rui-rong Chen, Pan-pan Ma, Fu-rong Liu, Sheng-ju Hao

**Affiliations:** ^1^ The Center for Medical Genetics in Gansu Provincial Maternity and Child-care Hospital, Gansu Provincial Clinical Research Center for Birth Defects and Rare Diseases, Lanzhou, China; ^2^ The Center for Reproductive Medicine in Gansu Provincial Maternity and Child-care Hospital, Lanzhou, China; ^3^ The Center for Medicine Imaging in Gansu Provincial Maternity and Child-care Hospital, Lanzhou, China

**Keywords:** nonketotic hyperglycinemia, glycine encephalopathy, high-throughput sequencing, AMT genes, glycine

## Abstract

**Background:** Nonketotic hyperglycinemia is a metabolic disease with autosomal recessive inheritance due to the glycine cleavage system (GCS) defect leading to the accumulation of glycine that causes severe and fatal neurological symptoms in the neonatal period.

**Methods:** Genomic DNA was extracted from the peripheral blood of the female proband and her family members. The *AMT* variation was detected in the patient by whole-exome sequencing (WES), and the variant was validated by Sanger sequencing.

**Results:** The WES showed that there were novel compound heterozygous frameshift variations c.977delA (p.Glu326Glyfs*12) and c.982_983insG (p.Ala328Glyfs*22) in exon eight of the *AMT* gene (NM_000481.4) in the proband. Genetic analysis showed that the former was inherited from the mother, and the latter was inherited from the father.

**Conclusion:** We report the novel compound heterozygous variation of the *AMT* gene in a Chinese girl with NKH by WES, which has never been reported previously. Our case expanded the *AMT* gene mutation spectrum, further strengthened the understanding of NKH, and deepened the genetic and clinical heterogeneity of the disease. However, the study of treatment and prognosis is still our future challenge and focus.

## Introduction

Nonketotic hyperglycinemia (NKH), also known as glycine encephalopathy (#OMIM: 605899), is an inherited disorder characterized by abnormally elevated glycine levels. It is usually caused by the defect of the enzyme that breaks down glycine in the body, which causes its accumulation in tissues and organs, especially the brain, which can cause severe nervous system damage. According to epidemiological statistics (Applegarth et al., 1979; Coughlin et al., 2017), the incidence of newborns is 1/55,000 in Finland and 1/63,000 in British Columbia, Canada. The clinical manifestations of NKH are different and can be divided into classic and non-classical types (Feng et al., 2021). The classic type is more common (84%), with progressive encephalopathy manifestations, low response, lethargy, hypotonia, vomiting, myoclonic epilepsy, and symptoms such as hiccups or apneas. And the symptoms worsen in a short period of time, and most children need ventilator support. Approximately 80% of NKH is caused by mutations in the *GLDC* gene (Bayrak et al., 2021). The *AMT* gene mutation causes approximately 20% of cases (Cao et al., 2021). In this study, whole-exome sequencing (WES) combined with Sanger sequencing technology was used to detect the molecular pathogenicity of the proband. The data of patients combined with clinical manifestations were analyzed to clarify the possible causes of the disease and provide a theoretical basis for their clinical diagnosis and genetic counseling.

## Materials and Methods

### Subject

**FIGURE 1:**
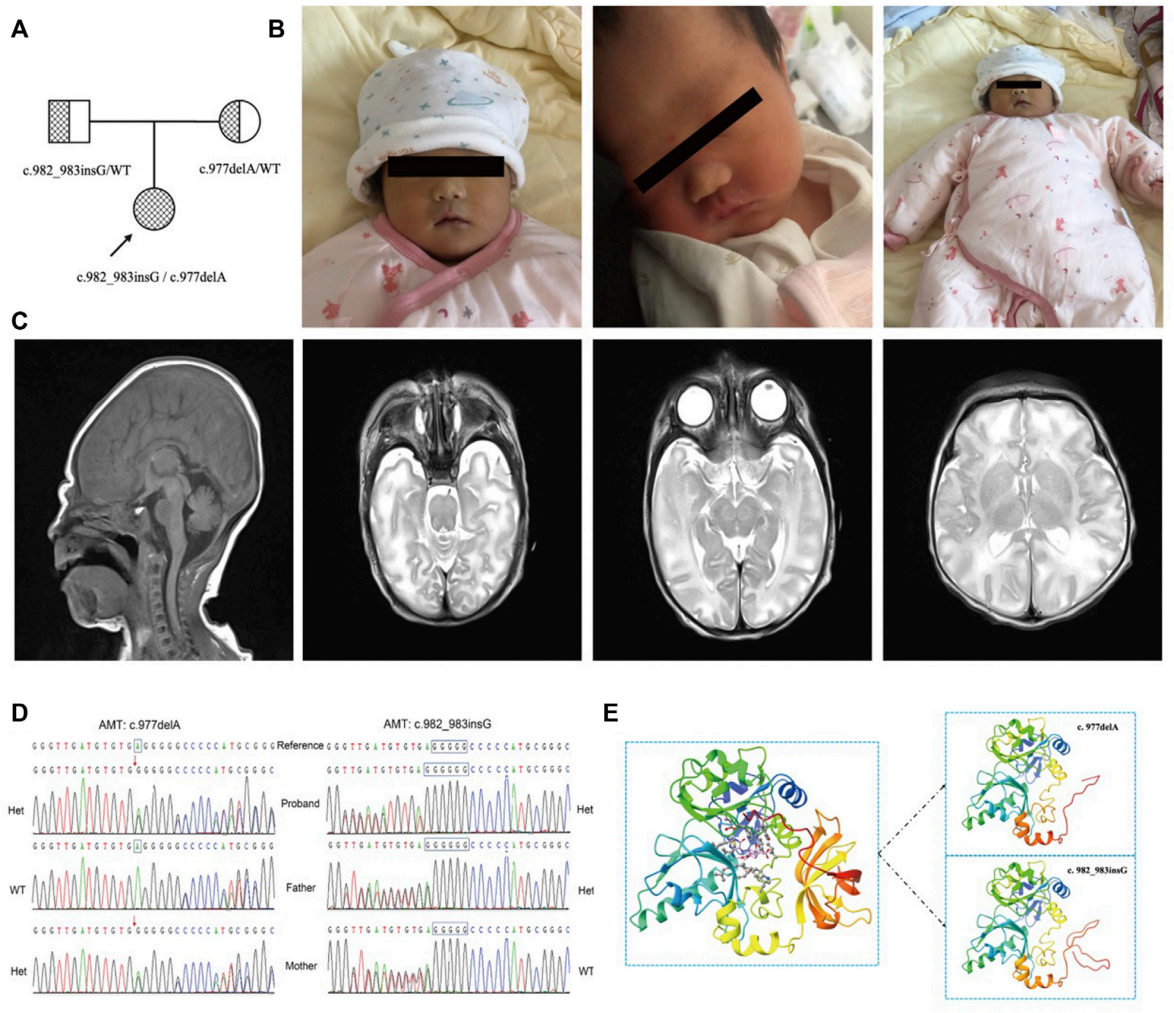
**(A)** The pedigree of the family. The proband is a compound heterozygous mutation in the *AMT* gene. Both of their parents are carriers. **(B)**. The image of the proband at birth. The complexion is ruddy, the reaction is good, the consciousness is clear, and the appearance is not obviously abnormal. **(C)**. The brain MRI of the proband. It shows that the hind limbs of the bilateral internal capsule, the midbrain, and the pons show symmetrical cytotoxic edema, and the bilateral globus pallidus in the T1WI sequence presents an increasing signal. **(D)**. The verification result of Sanger sequencing. The proband is a compound heterozygous variation with **(C)** 977delA and **(C)** 982_983insG of the *AMT* gene. The variant **(C)** 977delA is from the mother. **(C)** 982_983insG is from the father. Het is heterogenous, WT is wild type. **(E)** 3D image of AMT protein. The left of the figure is the 3D overall picture of wildtype (WT) AMT protein, the upper part of the right figure is the 3D overall picture of p.Glu326Glyfs*12 mutant (MUT) AMT protein, and the lower part of the right figure is the 3D overall picture of p.Ala328Glyfs*22 mutant (MUT) AMT protein.

The female pediatric patient was the first child of the parents (Figure 1A) and was born in Gansu Province Maternal and Child Health Hospital at 39 weeks of gestational age. The child’s birth weight was 2,970 g, and her Apgar score was nine at the first minute, 10 at the fifth minute, and 10 at the tenth minute after birth (Figure 1B). The patient had no premature rupture of membranes, low and clear amniotic fluid, an umbilical cord around the neck, and a pregnancy with a complete uterine mediastinum. The physical examination is as follows: body temperature is 36.8°C; pulse is 130 beats/min; and breathing is 45 beats/min. The complexion is ruddy, the reaction is good, the consciousness is clear, and the appearance is not obviously abnormal (Figure 1B). Two days after birth, the child had poor mental response, weak muscle tone, poor limb mobility, and poor spontaneous breathing. Blood oxygen saturation when monitored was about 70%. The tracheal intubation was urgently performed, and the balloon was pressurized to give oxygen. The chest radiograph showed that the texture of both lungs was thickened. After the child was assisted in breathing with the ventilator HFO mode (MAP: 10 cm H_2_O, amplitude: 22 cm H_2_O, frequency: 8 Hz, FIO2: 50%), the breathing gradually became stable. The blood test of the child showed that the percentage of neutrophils was 73.8%, the prothrombin activation time was 54 s, the cerebrospinal fluid chloride was 111 mmol/L, and the blood ammonia was 90.3 umol/L. Blood tandem mass spectrometry showed that glycine was 3084.08 umol/L (125-450 umol/L), and the ratio of glycine to phenylalanine was 48.97 (4.19–20.39). Brain MRI showed symmetrical cytotoxic edema of the hind limbs, midbrain, and pons on both sides of the internal capsule. Subdural hemorrhage on the posterior border of the right cerebellum should be considered. The signal of the bilateral pallidus on the T1WI sequence was slightly increased (Figure 1C). The final diagnosis was cerebral edema (brain stem), respiratory failure, and congenital genetic metabolic disease. Whole-exome sequencing was performed to define the diagnosis.

### Specimen Collection and Genomic DNA Extraction

After the patient’s family members signed an informed consent form for genetic testing, 2–5 ml of EDTA-K2 anticoagulated peripheral blood of the child and parents was collected. The Tiangen blood genomic DNA extraction kit (article number: DP329) was used to extract genomic DNA from patients and parents. NanoDrop 2000 is used for DNA concentration determination. The DNA concentration was 50–100 ng/ml, and the A260/280 ratio was maintained at 1.8–2.0.

### Whole Exome Sequencing and Bioinformatics Analysis

Protein-coding exome enrichment was performed using the xGen Exome Research Panel v2.0 (IDT, Iowa, United States) which consists of 429,826 individually synthesized and quality-controlled probes, which target 39 Mb of protein-coding region (19,396 genes) of the human genome and covers 51 Mb of end-to-end tiled probe space. Whole-exome sequencing (WES) was performed by using the MGISEQ-T7 series sequencer, and not less than 99% of the target sequences were sequenced. The sequencing process was performed by the Beijing Chigene Translational Medicine Research Center Co., Ltd., 100875, Beijing. Raw data were processed by fastp for adapters removing and low-quality reads filtering. The paired-end reads were performed using the Burrows-Wheeler Aligner (BWA) to the ensemble GRCh37/hg19 reference genome. Base quality score recalibration together with SNP and short indel calling was conducted using GATK. According to the sequencing depth and variant quality, SNPs and Indels were screened such that high-quality and reliable variants were obtained. The databases for minor allele frequencies (MAFs) annotation include 1,000 genomes, dbSNP, ESP, ExAC database; Provean, Sift, Polypen2_hdiv, Polypen2_hvar, Mutation taster, M-Cap, and REVEL software packages were used to predict protein product structure variation. As a prioritized pathogenicity annotation to the ACMG guideline, OMIM, HGMD, and ClinVar databases were used as conferences of pathogenicity for every variant. To predict the functional change of variants on the splicing sites, MaxEntScan, dbscSNV, and GTAG software packages were used instead of product structure prediction software.

### Sanger Sequencing Verification

After the analysis of the second-generation sequencing data, it was found that the pathogenic variant of the *AMT* gene was consistent with the cause of the family. Sanger sequencing was used for verification. The primers were designed by OLIGO 7 software. The upstream primer F was CTA​GTC​ACA​GTA​CCT​GTC​AAG​CAA and the downstream primer R was AAGGGAGGAATAGAG CCTGGAGTA. The fragment length was 304 bp. PCR reaction conditions: 95°C 3 min; (94°C 30 s, 60°C 45 s, 72°C 1 min) **×**32; 72°C 10 min, 4°C.

## Results

In this report, the compound heterozygous variation was found in the proband’s *AMT* gene, which was assessed as a pathogenic variation according to the latest guidelines and was newly developed. The paternal mutation site identified as c.982_983insG carries exon eight of the *AMT* gene [p. Ala328Glyfs*22]. According to the classification standards and guidelines of genetic variation of the American Society for Medical Genetics and Genomics (ACMG), this variation led to protein-truncating mutation, which might lead to nonsense-mediated mRNA decay (NMD) phenomenon, so PVS1 evidence was used. At the same time, the variation was not included in all normal population databases, and PM2 evidence could be used. Finally, the mutation was evaluated as a likely pathogenic mutation (LP). The patient’s other variant c.977delA [p. Glu326Glyfs*12] from the mother was also truncated, and PVS1 evidence could be used. This variation was also not included in any normal population database, and PM2 evidence could be used. And then, PM3 evidence was used when likely pathogenic mutations were detected at the transposition. The mutation was finally evaluated as pathogenic mutation (P). This compound heterozygous variation was verified by Sanger sequencing (Figure 1D). To obtain the theoretical structure of these gene mutants, 3D models were built on the public website SWISS-MODEL (Waterhouse et al., 2018) (https://swissmodel.expasy.org/; Figure 1E).

## Discussion

Nonketotic hyperglycinemia (NKH) has clinical heterogeneity, which is mainly divided into classic and non-classical types, and the classic type is more common (84%). The neonatal form of classic NKH involves genes that encode the glycine cleavage system and usually manifests as progressive encephalopathy from 6 h to 8 days after birth. The symptoms get worse in a short period of time, and most children need ventilator support (Kure et al., 1997; Chauke et al., 2016). Approximately 30% of the classical NHKs die in the neonatal period, and most of the children die within one year of age. Most of the survivors have severe brain development disorders and refractory epilepsy. We report a female patient with nonketotic hyperglycinemia, presenting with cerebral edema, respiratory failure, abnormal biochemical indicators including elevated glycine, elevated cerebrospinal fluid chloride, and abnormal MRI. This is caused by the compound heterozygous mutation in exon eight of the *AMT* gene, which has not been reported previously. The patient’s phenotype is classic glycine encephalopathy, which is onset in newborns, mainly due to the defect of amino methyltransferase in the glycine lyase system.

The metabolism of glycine is completed by the glycine lyase system (EC2.1.2.10) in the mitochondria in the body (Kure et al., 2001; Leung et al., 2020), which is composed of four protein components as follows: P protein (Kure et al., 2006), also known as pyridoxal phosphate-dependent glycine decarboxylase, which is produced by *GLDC* (OMIM: 238300) gene encoding; H protein (Poothrikovil et al., 2019), a lipoic acid-containing protein, encoded by the *GCSH* (OMIM: 238330) gene; T protein (Toone et al., 2003), also known as amino methyltransferase, is a kind of tetrahydrofolate-dependent transfer. The methyl enzyme is encoded by the *AMT* (OMIM: 238310) gene; the L protein (Bravo-Alonso et al., 2017), which is a lipoic acid dehydrogenase, is encoded by the *DLD* (OMIM: 238331) gene. It has been found that defects in T, P, and H proteins can lead to glycine encephalopathy. P protein defects are the most common, followed by T protein defects, and L protein defects are rare. The HGMD database includes 433 pathogenic mutation sites of P protein, 174 of which are clearly damaged by NKH; 98 pathogenic mutation sites of T protein are included, of which 44 are clearly damaged by NKH (Stenson et al., 2020). A compound heterozygous variant in the *AMT* gene of the proband was detected this time. It was assessed as a pathogenic variant according to the latest ACMG guidelines and was an unreported previous variant. There was an increase in glycine in the blood caused by a defect in the T protein of the glycine cleavage system. The inability to metabolize causes damage to the brain system, which can explain the clinical cause of the proband.

According to the results of genetic testing, the proband carries the paternal mutation site c.982_983insG in the eighth exon of the *AMT* gene, which is the G insertion between the 982nd and the 983rd position. Therefore, a frameshift occurs at the 328th amino acid of the protein sequence, so the translation is terminated prematurely at the 22nd amino acid position after that, resulting in protein truncation. According to the American Academy of Medical Genetics and Genomics (ACMG) genetic variation classification standards and guidelines (Richards et al., 2015; Abou Tayoun et al., 2018; Tavtigian et al., 2018), the variation is a protein-truncating mutation that may cause nonsense-mediated mRNA decay (NMD) phenomenon, so PVS1 is used as evidence. At the same time, if the mutation is not included in the gnomAD database (Karczewski et al., 2020), PM2 evidence can be used. Based on the evidence of PVS1 and PM2, this variant is assessed as likely pathogenic. The proband carries the maternal variant site c.977delA in the eighth exon of the *AMT* gene, which is the A deletion in the 977th position. The frameshift occurs at the 326th amino acid of the protein sequence, so the translation is terminated prematurely at the 12th amino acid position after that, resulting in protein truncation and forming a non-functional protein. Similarly, PVS1 evidence can be used; the gnomAD database does not include the mutation, and PM2 evidence can be used; at the same time, if the aforementioned possible pathogenic mutations are detected in the transposition, PM3 evidence can be combined with PVS1, PM2, and PM3 evidence, the variant is assessed as a pathogenic mutation (pathogenic).

Treatment of NKH is still achieved through symptomatic treatment (Korman et al., 2006; Magwebu et al., 2019). The use of low-dose strychnine nitrate and exchange blood therapy can reduce the blood glycine content, but these treatments have caused brain damage and further preventive treatments are invalid (von Wendt et al., 1980). Therefore, treatment is focused on reducing plasma glycine concentration by initiating sodium benzoate and utilizing N-methyl-D-aspartate receptor site antagonists (i.e., dextromethorphan and oral ketamine) to reduce glycinergic stimulation (Van Hove et al., 2005; Prasad et al., 2015). This therapy has been shown to be effective in controlling seizures and neurodevelopment in selected nonketotic hyperglycinemic populations (Bjoraker et al., 2016). So, if suspected NKH patients are found clinically, genetic diagnosis and corresponding treatment should be carried out as soon as possible so as not to delay the irreversible brain damage and bring a great burden to the whole family and society.

In conclusion, this study reports two novel compound heterozygous missense mutations in the *AMT* gene in the Han family of NKH: c.982dupG and c.977delA. The discovery and reporting of NKH-related gene mutations are helpful in analyzing genotypes. The correlation of phenotypes can clarify the etiology of patients. It provides a theoretical basis for clinical diagnosis and treatment and genetic counseling and expands the spectrum of pathogenic gene mutations in NKH. In addition, our research shows that whole-exome sequencing is very helpful for congenital disease screening, genetic diagnosis, and clinical genetic counseling.

## Data Availability

The original contributions presented in the study are included in the article/Supplementary Material; further inquiries can be directed to the corresponding author.
